# Neutrophil CD64 expression as a diagnostic marker for sepsis in adult patients: a meta-analysis

**DOI:** 10.1186/s13054-015-0972-z

**Published:** 2015-06-10

**Authors:** Xiao Wang, Zhong-Yun Li, Ling Zeng, An-Qiang Zhang, Wei Pan, Wei Gu, Jian-Xin Jiang

**Affiliations:** State Key Laboratory of Trauma, Burns and Combined Injury, Institute of Surgery Research, Daping Hospital, Third Military Medical University, Changjiang Road 10, Yuzhong District Chongqing, China; The First Affiliated Hospital of Wenzhou Medical University, Wenzhou, Zhejiang Province China; The 153 Central Hospital of PLA Jinan Military Region, Zhengzhou, Henan Province China

## Abstract

**Introduction:**

Neutrophil CD64 (nCD64) expression appears to be a promising marker of bacterial infections. The aim of this meta-analysis was to assess the accuracy of nCD64 expression for the diagnosis of sepsis in critically ill adult patients.

**Methods:**

We systematically searched PubMed, Embase, ISI Web of Knowledge, and the Cochrane Library for literature published between database inception and 19 May 2014, as well as reference lists of identified primary studies. Studies were included if they included assessment of the accuracy of nCD64 expression for sepsis diagnosis in adult patients and provided sufficient information to construct a 2×2 contingency table.

**Results:**

A total of 8 studies comprising 1986 patients fulfilled the inclusion criteria for the final analysis. The pooled sensitivity and specificity were 0.76 (95 % confidence interval [CI], 0.73–0.78) and 0.85 (95 % CI, 0.82–0.87), respectively. The positive likelihood ratio, negative likelihood ratio and diagnostic odds ratio were 8.15 (95 % CI, 3.82–17.36), 0.16 (95 % CI, 0.09–0.30), and 60.41 (95 % CI, 15.87–229.90), respectively. The area under the summary receiver operating characteristic curve of nCD64 expression with Q* value were 0.95 (Q* =0.89).

**Conclusions:**

On the basis of our meta-analysis, nCD64 expression is a helpful marker for early diagnosis of sepsis in critically ill patients. The results of the test should not be used alone to diagnose sepsis, but instead should be interpreted in combination with medical history, physical examination, and other test results.

## Introduction

Sepsis is the most common cause of mortality in critically ill patients worldwide [1]. Delays in diagnosis and treatment often result in rapid progression to circulatory collapse, multiple organ failure, and eventually death [2]. Therefore, early diagnosis sepsis and timely treatment can improve patients’ outcome and reduce costs [3, 4].

The diagnosis of sepsis is sometimes challenging, because the diagnosis is based on systemic inflammatory response syndrome (SIRS) in the presence of a known infection. SIRS is very common in many conditions, such as surgery, trauma, and pancreatitis [5, 6]. Microbiological culture is a gold standard for distinguishing sepsis from non-infectious conditions. However, incubation of bacteria may take a long time, and during this period, the condition of patients may rapidly deteriorate. Additionally, blood culture always has poor sensitivity [7]. Thus, there is an urgent need of a biomarker that can identify sepsis in an early stage so that timely and appropriate use of antibiotics can be initiated [8].

CD64, one of the high-affinity immunoglobulin Fcγ receptors, is constitutively expressed on monocytes and eosinophils. Recently, an increasing number of studies have been performed to investigate the role of neutrophil CD64 (nCD64) expression in the diagnosis of bacterial infection and sepsis [9–22]. Davis et al. indicated that nCD64 expression could improve the accuracy of diagnosing infection or sepsis [9]. Cardelli et al. reported that nCD64 expression had higher sensitivity and specificity than procalcitonin (PCT) in detecting sepsis [10]. However, these studies had limited numbers of patients and conflicting results [11–13]; thus, no firm conclusions could be drawn.

The authors of some meta-analyses have investigated the accuracy of nCD64 expression for the diagnosis of bacterial infection [23–25]. However, these studies included adults, children, and neonates, and patients with rheumatoid arthritis, local infections, and sepsis were mixed. None of these studies specially investigated the ability of nCD64 to diagnose sepsis in critically ill patients. Additionally, many more related studies have been published during the last 2 years. We aimed to conduct a meta-analysis to investigate the role of nCD64 expression for sepsis diagnosis in critically ill adult patients.

## Methods

A protocol was designed before this study was undertaken, as recommended by the Quality of Reporting of Meta-analyses statement [26]. All analyses are based on previously published studies; thus, neither ethical approval nor patient consent was required.

### Search strategy and selection criteria

We systematically searched PubMed, Embase, ISI Web of Knowledge, and the Cochrane Library to identify all studies that included assessment of the accuracy of nCD64 expression for the diagnosis of sepsis. Our search terms were “(CD64 OR “Fc gamma receptor”) AND (sepsis OR “septic shock” OR septicemia).” We searched the databases for literature published between database inception and 19 May 2014. Additionally, the reference lists of each primary study identified, as well as previous review articles, were hand-searched to identify other potentially eligible studies.

Eligibility of a study for the meta-analysis was based on the following selection criteria: assessed the diagnostic accuracy of nCD64 expression for sepsis; had a well-defined reference standard for sepsis, which included the use of accepted definitions by the American College of Chest Physicians/Society of Critical Care Medicine [27]; and provided sufficient information to construct a 2×2 contingency table. We included only publications written in English. Studies conducted on special groups of neonates and those that included patients who did not have SIRS or were not critically ill were excluded. Two investigators (XW and ZYL) reviewed all the studies independently. Disagreements were resolved through discussion with a third investigator (AQZ).

### Data extraction

Two investigators (XW and ZYL) reviewed all eligible studies and carefully extracted data. The data extracted from each study included the following details: first author, publication year, country of origin, clinical setting, patient demographics, sample size, analytical method, cutoff value, sensitivity, and specificity. Each reviewer extracted the data to construct a 2×2 contingency table. If there was any disagreement between the two reviewers, it was resolved by referral to a third investigator (AQZ). We contacted the authors of the selected articles by email if further information was needed. If there was no response, the study was excluded.

### Quality assessment

Two investigators (XW and ZYL) independently evaluated the methodological quality of each study by applying with the diagnostic accuracy tool Quality Assessment of Diagnostic Accuracy Studies (QUADAS) [28], which is recommended by the Cochrane Collaboration for the quality assessment of diagnostic studies. The QUADAS tool is constituted of a list of 14 questions: representative spectrum, clear description of study criteria, acceptable reference standard, disease progression bias avoided, partial verification bias avoided, differential verification bias avoided, incorporation bias avoided, detailed description of index test, detailed description of reference standard, blinding of investigators to reference, blinding of investigators to index test, availability of clinical data, uninterpretable results, and withdrawals explained. Questions with “yes”, “no,” and “unknown” answers were scored as 1, −1, and 0, respectively.

### Statistical analysis

The sensitivity, specificity, positive likelihood ratio (PLR), negative likelihood ratio (NLR), and diagnostic odds ratio (DOR) with corresponding 95 % confidence intervals (CI) were calculated for each study. Meanwhile, the pooled sensitivity, specificity, PLR, NLR, and DOR were also calculated for each group by using a bivariate meta-analysis model. The likelihood ratio expresses the magnitude by which the probability of sepsis in a given patient is modified by the results of the CD64 expression. The DOR is the ratio of the odds of a positive result in a patient with sepsis compared with a patient without sepsis: [sensitivity/(1 − sensitivity)]/[(1 − specificity)/specificity]. We also constructed summary receiver operating characteristic (SROC) curves, plotting sensitivity versus specificity, to illustrate the diagnostic accuracy. The area under the curve (AUC) with Q* value was also calculated.

We used Cochran’s Q test and the *I*^2^ statistic to evaluate the heterogeneity among the studies. In general, significant heterogeneity was considered when the *p* value was less than 0.05 and the *I*^2^ value was greater than 50 %. If there was significant heterogeneity, we chose a fixed model; if there was no heterogeneity, we chose a random model. Publication bias was examined by funnel plot and Egger test. All statistical analyses were performed using Meta-DiSc (version 1.4) and STATA (version 12.0; StataCorp, College Station, TX, USA) software.

## Results

### Study characteristics

**Fig. 1:**
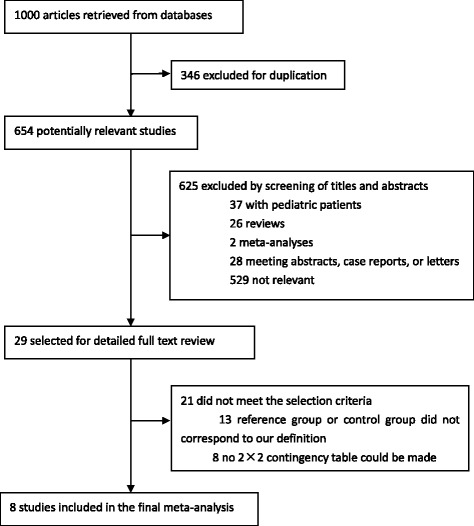
Flow diagram of the study selection process

In our database search, we retrieved 1000 articles, of which 971 were eliminated for various reasons based on the title and/or abstract. After full-text reviews, we excluded a further 21 studies: 13 studies’ reference group or control group did not correspond to our criteria; 8 studies did not report sufficient data to construct the 2×2 contingency table. Ultimately, eight studies fulfilled all eligibility criteria and were included in the final pooled analysis (Fig. 1). Searches of the reference lists did not identify any additional relevant articles.

The characteristics and data of each included study are listed in Table 1. The eight included trials were published between 2008 and 2014. The majority of the trials were performed in Europe; another two were done in Asia and South American. A total of 1986 critically ill patients were included, comprising 1376 patients from intensive care units (ICUs) in 7 studies [10, 11, 13–17] and 610 patients from emergency departments in 1 study [12]. Mean patient ages varied between 51 and 72 years, and the proportion of men included ranged from 48 % to 72 %. Among 1986 patients, 1002 had sepsis. Five studies used flow cytometry (FCM) to detect nCD64 expression. Two studies used the Leuko64 kit (Trillium Diagnostics, Brewer, ME, USA), and one study used hematology analyzers. All the included studies had calculated the optimal cutoff point based on the ROC curve. However, we did not obtain a consistent result. The methodological quality of all included studies was appraised by using the QUADAS tool, and the results are shown in Table 1.

**Table 1:** Main characteristics of the included studies

Study	Year	Country	Sepsis definition	Sepsis diagnosis	Study population	Assay method	Cutoff	AUC	*n*	TP	FP	FN	TN	QUADAS
Dimoula et al. [11]	2014	Belgium	ISDC^a^	Clinical or proven^b^	Cases: patients with sepsis in ICU	FCM	MFI 230	0.94	468	92	47	11	318	11
					Controls: SIRS patients without sepsis in ICU									
Righi et al. [17]	2014	Italy	ISDC	Clinical or proven	Cases: patients with sepsis in ICU	FCM	2000 ABC	0.93	93	55	1	6	31	8
					Controls: patients without sepsis in ICU									
Gerrits et al. [13]	2013	Netherlands	ISDC	Proven	Cases: patients with sepsis in ICU	Hematology analyzer	1.66	NR	44	25	1	0	18	10
					Controls: SIRS patients without sepsis in ICU									
Gros et al. [15]	2012	France	ISDC	Proven	Cases: patients with sepsis in ICU	Leuko64 kit	2.2	0.80	293	93	16	55	129	10
					Controls: SIRS patients without sepsis in ICU									
Gibot et al. [14]	2012	France	ISDC	Clinical or proven	Cases: patients with sepsis in ICU	FCM	1.62	0.95	300	130	7	24	139	11
					Controls: patients without sepsis in ICU									
Hsu et al. [16]	2011	China	ISDC	Clinical or proven	Cases: patients with sepsis in ICU	FCM	4300 molecules/cell	0.93	66	49	1	6	10	9
					Controls: SIRS patients without sepsis in ICU									
Gámez-Díaz et al. [12]	2011	Colombia	ISDC	Clinical or proven	Cases: patients with sepsis in ED	Leuko64 kit	1.7	0.71	610	266	73	138	133	9
					Controls: patients without sepsis in ED									
Cardelli et al. [10]	2008	Italy	ISDC	Proven	Cases: patients with sepsis in ICU	FCM	2398 molecules/cell	0.97	112	50	5	2	55	9
					Controls: patients without sepsis in ICU									

*Abbreviations: ABC* antibody-binding capacity, *AUC* area under the curve, *ED* emergency department, *FCM* flow cytometry, *ICU* intensive care unit, *ISDC* International Sepsis Definition Conference, *MFI* mean fluorescence intensity, *QUADAS* Quality Assessment of Diagnostic Accuracy Studies, *SIRS* systemic inflammatory response syndrome, *TP* true positive, *FP* false positive, *FN* false negative, *TN* true negative, *NR* not reported

^a^2001 SCCM/ESICM/ACCP/ATS/SIS International Sepsis Definitions Conference [27]

^b^ *Clinical infection* was defined as infection suspected on a clinical basis. *Proven infection* was defined as culture-proven infection with an identified microorganism

### Quantitative data synthesis

**Fig. 2:**
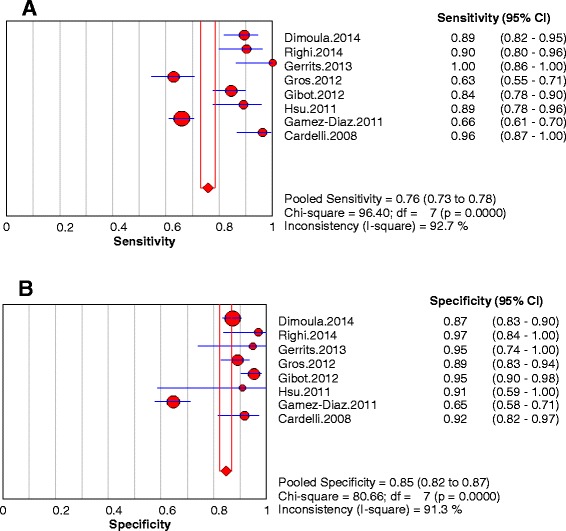
Forest plots of the sensitivity (**a**) and specificity (**b**) of neutrophil CD64 of all included studies. The *solid circles* represent each individual study, and the *solid diamonds* represent the pooled diagnostic odds ratios. The sizes of the circles are proportional to the sizes of the included studies. Error bars are 95 % confidence intervals (CIs)

**Fig. 3:**
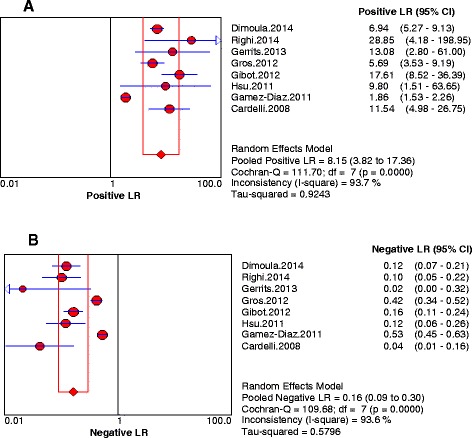
Forest plots of the positive likelihood (**a**) and negative likelihood (**b**) ratios (LRs) of neutrophil CD64 of all included studies. The *solid circles* represent individual studies, and the *solid diamonds* represent the pooled diagnostic odds ratios. The sizes of the circles are proportional to the sizes of the included studies. Error bars are 95 % confidence intervals (CIs)

**Fig. 4:**
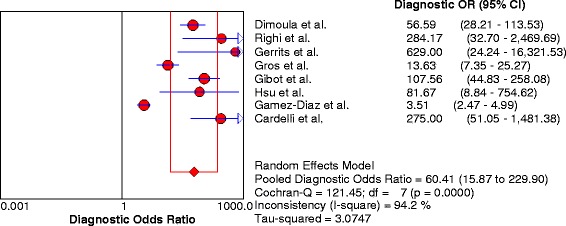
Summary diagnostic odds ratios (OR) of neutrophil CD64 of all included studies. The *solid circles* represent individual studies, and the solid diamond represents the pooled diagnostic OR. The size of each circle is proportional to the size of the corresponding included study. Error bars are 95 % confidence intervals (CIs)

**Fig. 5:**
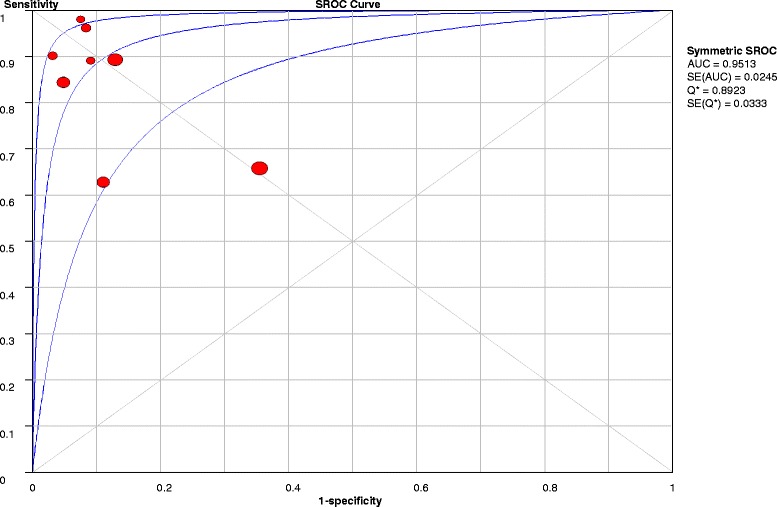
Summary receiver operating characteristic (SROC) curve of all included studies. The *circles* represent individual studies in the meta-analysis. The sizes of the circles are proportional to the size of the corresponding included study. *Abbreviations: AUC* area under the curve, *SE* standard error

The pooled sensitivity was 0.76 (95 % CI, 0.73–0.78) and pooled specificity was 0.85 (95 % CI, 0.82–0.87) (Fig. 2). The pooled PLR was 8.15 (95 % CI, 3.82–17.36), and the pooled NLR was 0.16 (95 % CI, 0.09–0.30) (Fig. 3). The SDOR was 60.41 (95 % CI, 15.87–229.90) (Fig. 4). The area under the SROC of nCD64 expression was 0.95, and the Q* value was 0.89, indicating a high level of diagnostic accuracy (Fig. 5).

We performed a subgroup analysis of five studies that used FCM to detect nCD64 expression. The pooled sensitivity, specificity, PLR, NLR, and SDOR of nCD64 were 0.88 (95 % CI, 0.85–0.92), 0.90 (95 % CI, 0.86–0.94), 11.56 (95 % CI, 5.92–22.60), 0.13 (95 % CI, 0.09–0.17), and 93.57 (95 % CI, 52.88–165.55), respectively. The area under the SROC of nCD64 expression was 0.96, and the Q* value was 0.91. It also displayed good diagnostic accuracy.

### Investigation of heterogeneity

We used the Cochran Q test and the *I*^2^ statistic to evaluate the presence of statistical heterogeneity. Significant heterogeneities were found for the pooled sensitivity, specificity, PLR, NLR, and SDOR. So, we performed a threshold analysis to explore the effect (Spearman correlation coefficient = −0.50, *p* =0.21) and found there was no statistically significant difference.

### Publication bias

Deeks funnel plot asymmetry and the results of the Egger test (*p* =0.02) suggested potential publication bias.

## Discussion

Sepsis is the most common cause of death in critically ill patients. A missed diagnosis of sepsis may result in a substantial delay in treatment, which may contribute to the high mortality. Therefore, clinicians often tend to prescribe antibiotics to reduce the risk of bacterial infections and sepsis. However, giving antibiotics to patients who have no bacterial infection is not necessary. Excessive use of antibiotics brings huge economic burden to society and contributes to the development of antibiotic resistance. So, early diagnosis and timely treatment of sepsis are vital to improving outcomes and lightening the financial burden for patients. Therefore, a diagnostic marker with high sensitivity and specificity for sepsis is urgently needed.

The quantification of nCD64 expression has recently been reported to be a useful biomarker for bacterial infection and sepsis. CD64 expression is low on resting neutrophils, and it is rapidly deregulated after activation [29–31]. When the stimulations are removed, nCD64 expression will dramatically decrease within 48 hours and be back to normal levels within 7 days [32, 33]. Additionally, nCD64 expression is relatively stable in blood samples for more than 30 hours, and the assay method is accurate, fast, and simple [34]. Most hospitals have equipment that can detect nCD64 expression. Moreover, nCD64 expression represents a pathophysiologic process that plays a key role in the innate immune response: neutrophils acting as phagocytes [35]. Therefore, nCD64 is one of the most useful markers for bacterial infections and sepsis.

In our study, we included only studies in which researchers assessed the accuracy of nCD64 for sepsis differentiation between critically ill patients with sepsis from those without sepsis. If the study researchers selected healthy volunteers, we excluded the study because healthy people rarely develop sepsis. Inclusion of healthy volunteers would lead to an overestimation of the overall diagnostic accuracy. Eventually, eight studies were included in our meta-analysis, and our results indicated that nCD64 expression plays an important role in diagnosing sepsis. We used a bivariate random-effects model, and the pooled sensitivity and specificity were 0.75 and 0.86, respectively. The pooled analysis showed that nCD64 seemed to have high degree of diagnostic accuracy for sepsis: The area under the SROC curve was 0.95, and the Q* value was 0.89. DOR is a single indicator of test accuracy and is independent of disease prevalence. The value of the DOR ranges from 0 to infinity, with higher values indicating greater diagnostic accuracy [36]. In the included studies, the DOR ranged from 3.51 to 629.00, and the pooled DOR was 60.41, indicating a high level of overall accuracy. The PLR and the NLR are considered to be more clinically meaningful measures of diagnostic accuracy. In this study, the pooled PLR was 8.15, and the pooled NLR was 0.16. FCM is the most common method for detecting nCD64. So, we did a subgroup analysis. It showed that detecting nCD64 by FCM also had a good diagnostic accuracy. The pooled sensitivity, specificity, PLR, NLR, DOR, and AUC were 0.88, 0.90, 11.56, 0.13, 93.57, and 0.96, respectively. Taken together, these data suggested that nCD64 expression could be a very useful marker for early diagnosis of sepsis and that FCM might be the ideal assay method for detecting nCD64 expression.

The authors of three prior meta-analyses assessed the role of nCD64 expression for diagnosing bacterial infection. Cid et al. and Li et al. concluded that nCD64 expression is a reliable biomarker for the early diagnosis of bacterial infection [23, 25]. Jia et al. concluded that nCD64 expression can be used as an additional test in the diagnosis of neonatal infection [24]. However, the calculations in these meta-analyses were performed using studies with highly variable designs: adults, children, and neonates were included, and patients with bacterial infection, local infection, and sepsis were mixed. Sepsis is different from local infection, and adult sepsis is different from neonatal sepsis. Therefore, the results of these meta-analyses did not sufficiently indicate that nCD64 is a useful marker of sepsis. In our present meta-analysis, we assessed the diagnostic accuracy of nCD64 for sepsis and focused only on the adult patients.

The eight included studies also included assessments of the diagnostic accuracy of PCT, CRP, soluble triggering receptor expresses on myeloid cells 1 (sTREM-1), and other markers. The results of those studies all showed that nCD64 had better accuracy [10–17]. However, other meta-analyses indicated that the pooled sensitivity and specificity were 0.77 and 0.86, respectively, for PCT and 0.79 and 080, respectively, for sTREM-1 [37, 38]. It was difficult to evaluate which marker was better on the basis of the meta-analysis results.

As our results show that nCD64 is not a perfect marker for sepsis, but an ideal marker does not exist, because sepsis is a complex, dynamic syndrome and no single test is sufficiently sensitive and specific for detecting sepsis. As yet, we have not found a biomarker with sufficient (>0.9) sensitivity and specificity to diagnose sepsis. However, an increasing number of studies have indicated that combinations of various markers are a useful approach to improving the accuracy of diagnosing sepsis [39, 40]. Gibot et al. indicated that a combination of nCD64, sTREM-1, and PCT could have a far better diagnostic performance for sepsis, with an AUC of 0.97 [14]. Nevertheless, nCD64 is one of the most promising parameters. The diagnosis accuracy of nCD64 will be confirmed as research continues. If these studies give the expected positive results, nCD64 will become a routine parameter for ICU patients. This will improve antibiotic management, reduce antibiotic resistance, and reduce mortality.

Our meta-analysis has several limitations. First, this meta-analysis included only eight studies, though we did our best to search eligible studies. One reason for this may be that we included only publications written in English. Second, the methodological quality of each study was acceptable. However, all the included studies could not completely meet the standards of QUADAS. Third, we detected significant heterogeneity between studies. Generally, the threshold effect is a very common source of heterogeneity in a diagnostic study, but we did not find significant differences in this regard. The studies differed in several ways (e.g., patients’ clinical spectrum, age, sex, admission category, and nCD64 assay used). All these differences probably contributed to the heterogeneity. However, the meta-analysis included only eight studies, and we did not do a meta-regression to explore the source of the heterogeneities. The heterogeneity seriously affected the accuracy of our results. Fourth, the included studies used different criteria to diagnose infection. In some studies, infection was diagnosed by microbiological culture, and in others the diagnosis was made on a clinical basis. Fifth, we detected publication bias. Studies with positive results were more likely to be published, and studies with negative results were rarely published. This led to an overestimation of the overall diagnostic accuracy. Sixth, we could not determine the ideal cutoff point for the nCD64 test, because there were several assay methods for nCD64 test and we did not have enough data. Finally, some of the studies indicated that nCD64 could reflect the severity and prognosis of sepsis [41, 42], but we did not consider this issue.

## Conclusions

Although our meta-analysis has various limitations exist, it suggests that nCD64 expression is a helpful marker for early diagnosis of sepsis in critically ill adult patients. However, nCD64 expression is not sufficient to correctly distinguish all patients with sepsis from critically ill patients. It must be interpreted in combination with medical history, physical examination, and other test results. Before the CD64 test is widely used in the clinical setting, we need further larger, multicenter studies to confirm its predictive value.

## Key messages

- We lack an ideal biomarker for early diagnosis of sepsis.
- The level of CD64 expression on neutrophils is associated with bacterial infection and sepsis.
- Flow cytometry is the most common method for detecting nCD64.
- nCD64 expression is a helpful biomarker for the early diagnosis of sepsis.
- We need multimarker panels to improve diagnostic accuracy for sepsis.

## Abbreviations

ABC: Antibody binding capacity
AUC: Area under the curve
CI: Confidence interval
DOR: Diagnostic odds ratio
ED: Emergency department
FCM: Flow cytometry
ICU: Intensive care unit
ISDC: International Sepsis Definition Conference
MFI: Melt flow index
nCD64: Neutrophil CD64
NLR: Negative likelihood ratio
PCT: Procalcitonin
PLR: Positive likelihood ratio
QUADAS: Quality Assessment of Diagnostic Accuracy Studies
ROC: Receiver operating characteristic
SDOR: Summary diagnostic odds ratio
SIRS: Systemic inflammatory response syndrome
SROC: Summary receiver operating characteristic curve
sTREM-1: Soluble triggering receptor expresses on myeloid cells 1
