# Erratum to: ‘Neutrophil CD64 expression as a diagnostic marker for sepsis in adult patients: a meta-analysis’

**DOI:** 10.1186/s13054-016-1357-7

**Published:** 2016-06-03

**Authors:** Xiao Wang, Zhong-Yun Li, Ling Zeng, An-Qiang Zhang, Wei Pan, Wei Gu, Jian-Xin Jiang

**Affiliations:** State Key Laboratory of Trauma, Burns and Combined Injury, Institute of Surgery Research, Daping Hospital, Third Military Medical University, Chongqing, China; The First Affiliated Hospital of Wenzhou Medical University, Wenzhou, China; The 153 Central Hospital of PLA Jinan Military Region, Zhengzhou, China

Unfortunately, the original version of this article [1] contained an error. A sentence in the statistical analysis section was written incorrectly. It was written wrongly as:

“If there was significant heterogeneity, we choose a fixed model; if there was no heterogeneity, we choose a random model.”

The correct form of this sentence is:

“If there was significant heterogeneity, we choose a random model; if there was no heterogeneity, we choose a fixed model.”
